# A new species of *Miraphaedusa* Nordsieck, 2005 (Gastropoda, Stylommatophora, Clausiliidae) from Guizhou, China

**DOI:** 10.3897/BDJ.13.e165827

**Published:** 2025-10-03

**Authors:** Zhong-Guang Chen, Yu-Ting Dai, Yi-Jun Li, Jiao Jiang, Xiao-Ping Wu, Yi-Feng Fang, Shan Ouyang

**Affiliations:** 1 School of Life Sciences, Nanchang University, Nanchang, China School of Life Sciences, Nanchang University Nanchang China; 2 College of Animal Science and Technology, Jiangxi Agricultural University, Nanchang, China College of Animal Science and Technology, Jiangxi Agricultural University Nanchang China; 3 School of geoscience, faculty of science, the University of Sydney, Sydney, Australia School of geoscience, faculty of science, the University of Sydney Sydney Australia; 4 Zhejiang Museum of Natural History, Hangzhou, China Zhejiang Museum of Natural History Hangzhou China

**Keywords:** land snail, taxonomy, clausiliids, conchology, Phaedusinae

## Abstract

**Background:**

The genus *Miraphaedusa* Nordsieck, 2005 is a group of medium to large clausiliid land snails endemic to Guangxi and Guizhou Provinces of China and consists of three species. It is characterised by a slender to fusiform shell with thick apical part, strongly doubled peristome and several short palatal plicae.

**New information:**

A new species, *Miraphaedusa xihuashida* Chen sp. nov., is described from Tongren, Guizhou, China. It is characterised by the fusiform shell with 10.5–12.25 whorls and 24.7–27.5 mm shell height, thick, expanded, reflexed and strongly doubled peristome, weak inferior and subcolumellar lamella and short, lateral palatal plicae.

## Introduction

Southern China is renowned for its exceptionally diverse fauna of clausiliid land snails (Nordsieck 2001, Nordsieck 2003, Nordsieck 2005, Nordsieck 2007, Nordsieck 2010, Grego and Szekeres 2011, Nordsieck 2012a, Nordsieck 2012b, Nordsieck 2012c, Hunyadi and Szekeres 2016, Nordsieck 2016, Grego and Szekeres 2017, Grego and Szekeres 2019, Grego and Szekeres 2020, Nordsieck 2021). In recent years, a large number of new clausiliid species have been continuously discovered in this region (Hunyadi and Szekeres 2016, Nordsieck 2016, Grego and Szekeres 2017, Grego and Szekeres 2019, Do et al. 2019, Chen 2020, Grego and Szekeres 2020, Chen 2021, Chen and Ouyang 2021, Qiu 2021, Chen 2022, Qiu 2022, Chen et al. 2024a, Chen et al. 2024b, Xu et al. 2024). The species diversity of Clausiliidae here still remains to be explored.

The genus *Miraphaedusa* Nordsieck, 2005 of the family Clausiliidae Gray, 1855 is a group of medium to large land snails, characterised by their slender to fusiform shell with thick apical part, the strongly doubled peristome and several short palatal plicae (Nordsieck 2005, Grego and Szekeres 2011, Nordsieck 2012a, Hunyadi and Szekeres 2016). This genus, confined to a small distribution area in Guangxi and Guizhou, consists of only three species: *M. takagii* Nordsieck, 2005, *M. pretiosa* Nordsieck, 2012 and *M. gregoi* Hunyadi & Szekeres, 2016 (Nordsieck 2005, Grego and Szekeres 2011, Nordsieck 2012a, Hunyadi and Szekeres 2016).

In this study, we describe a new species of *Miraphaedusa* from Tongren, Guizhou, China, based on shell and genital morphology. Its discovery significantly expands the northern limit of the distribution of the genus.

## Materials and methods

Specimens were collected from Tongren, Guizhou, China in 2025. Living specimens were placed into boiling water for 10 seconds to take out the soft part, which was then preserved in 95% ethanol separately. Soft parts were softened by soaking in 20% ethanol for 10 minutes before dissection. Empty shells were cleaned, dried and preserved at room temperature. Measurements were taken with digital callipers to the nearest 0.1 mm. Whorls were counted as described by Kerney and Cameron (1979). In the description of the genitalia, the terms proximal and distal relate to the atrium. Photographs were taken by an A7C II camera (Sony, Minato City, Japan) and modified in Adobe Photoshop CC 2015 (Adobe Systems, San Jose, US). Maps were made in ArcGIS Pro (Esri, Redlands, US).

Abbreviations: NCUMB: Museum of Biology, Nanchang University (Nanchang, Jiangxi, China); ZMNH: Zhejiang Museum of Natural History (Hangzhou, Zhejiang, China); SZ: collection of Miklós Szekeres (Budapest, Hungary).

## Taxon treatments

### Miraphaedusa xihuashida

Chen sp. nov.

30E28FBC-8B73-58C3-AA18-2C11EF09509E

1F0D7C93-B264-4385-ABD7-0DCCECA049A5

#### Materials

- **Type status:** Holotype. **Occurrence:** individualID: NCUMB 2507007; individualCount: 1; occurrenceID: F8F0D039-959A-5C70-A400-54F1BCC33D3A; **Taxon:** kingdom: Animalia; phylum: Mollusca; class: Gastropoda; order: stylommatophora; family: Clausiliidae; genus: Miraphaedusa; **Location:** country: China; stateProvince: Guizhou; municipality: Tongren; locality: Tongren Grand Canyon [铜仁大峡谷]; verbatimLatitude: 27.8825°N; verbatimLongitude: 109.2494°E; **Identification:** identifiedBy: Z.-G. Chen; **Event:** eventDate: June 2025; **Record Level:** institutionID: Museum of Biology, Nanchang University
- **Type status:** Paratype. **Occurrence:** individualID: NCUMB 2507008–10; individualCount: 3; occurrenceID: 54EA104A-D052-50A1-9DB6-B2CB0840AD6F; **Taxon:** kingdom: Animalia; phylum: Mollusca; class: Gastropoda; order: stylommatophora; family: Clausiliidae; genus: Miraphaedusa; **Location:** country: China; stateProvince: Guizhou; municipality: Tongren; locality: Tongren Grand Canyon [铜仁大峡谷]; verbatimLatitude: 27.8825°N; verbatimLongitude: 109.2494°E; **Identification:** identifiedBy: Z.-G. Chen; **Event:** eventDate: June 2025; **Record Level:** institutionID: Museum of Biology, Nanchang University
- **Type status:** Paratype. **Occurrence:** individualID: ZMNH AIMS29006; individualCount: 1; occurrenceID: 2D396C96-D3B0-5BC9-8DA5-3F646A888B51; **Taxon:** kingdom: Animalia; phylum: Mollusca; class: Gastropoda; order: stylommatophora; family: Clausiliidae; genus: Miraphaedusa; **Location:** country: China; stateProvince: Guizhou; municipality: Tongren; locality: Tongren Grand Canyon [铜仁大峡谷]; verbatimLatitude: 27.8825°N; verbatimLongitude: 109.2494°E; **Identification:** identifiedBy: Z.-G. Chen; **Event:** eventDate: June 2025; **Record Level:** institutionID: Zhejiang Museum of Natural History
- **Type status:** Paratype. **Occurrence:** individualID: SZ 25062506; individualCount: 2; occurrenceID: F1D3F180-D54D-5B3E-8902-6237E785E436; **Taxon:** kingdom: Animalia; phylum: Mollusca; class: Gastropoda; order: stylommatophora; family: Clausiliidae; genus: Miraphaedusa; **Location:** country: China; stateProvince: Guizhou; municipality: Tongren; locality: Tongren Grand Canyon [铜仁大峡谷]; verbatimLatitude: 27.8825°N; verbatimLongitude: 109.2494°E; **Identification:** identifiedBy: Z.-G. Chen; **Event:** eventDate: June 2025; **Record Level:** institutionID: Collection of Miklós Szekeres

#### Description

Shell (Fig. 1a). Shell fusiform, sinistral, thick-walled, solid, reddish-brown, with 10.5–12.25 whorls. Whorls have an opaque surface with thin and dense growth lines. Apical part thick. Aperture projected, narrow, oblong. Peristome strongly doubled, thick, expanded and reflexed, with or without indistinct palatal serration. Superior lamella strong, highest at the position near its outer end, extending to the peristome margin. Weak inferior and subcolumellar lamella closely positioned, the former descending to near to the peristome margin and the latter to the peristome margin. Principal plica long, initiates ventrally and ends near the aperture. Palatal plicae 4–5, short, lateral, nearly straight or slightly curved. Clausilium plate invisible through the aperture in oblique view. Measurements: holotype, shell height 27.5 mm, shell width 6.2 mm, aperture height 6.2 mm, aperture width 4.8 mm; paratypes, shell height 24.7–26.8 mm, shell width 5.8–6.2 mm, aperture height 5.8–6.2 mm, aperture width 4.4–4.7 mm.

**Figure 2.**
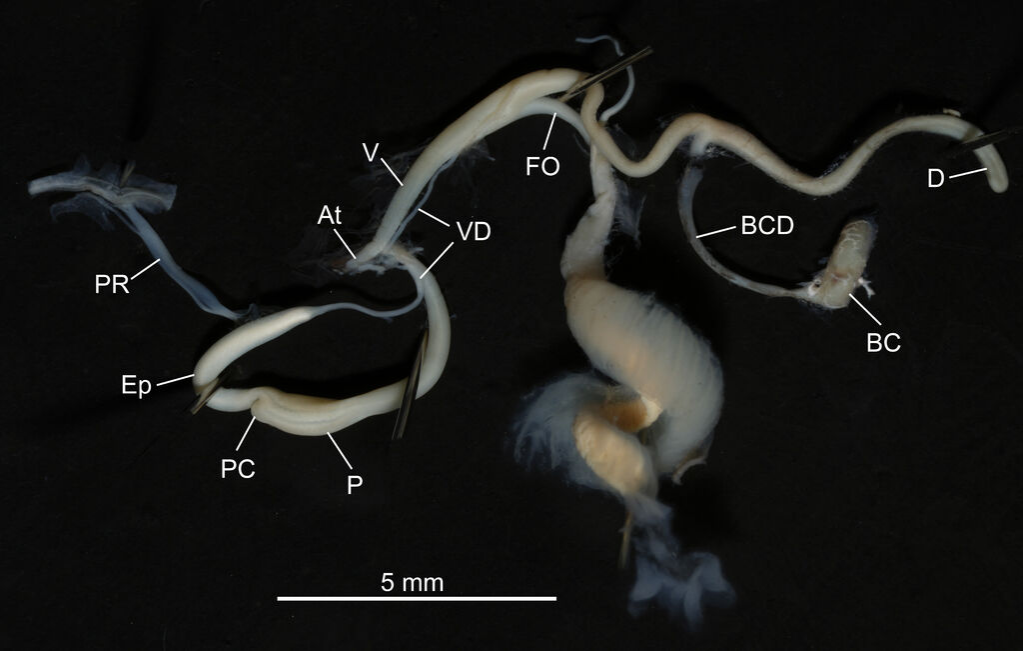
Genitalia of *Miraphaedusa xihuashida* Chen, sp. nov. Abbreviations: At: atrium; BC: bursa copulatrix; BCD: bursa copulatrix duct; D: diverticle of the bursa copulatrix duct; Ep: epiphallus; FO: free oviduct; P: penis; PC: penial caecum; PR: retractor muscle of the penial branch; V: vagina; VD: vas deferens.

Genitalia (Fig. 2). Atrium short and thin. Penis, cylindrical, gradually thickening distally, becoming narrower shortly before the transition to epiphallus. Penial caecum present. Epiphallus thick, shorter than penis. Penial retractor long and thick, inserted at the middle part of epiphallus. Vas deferens relatively thin and short. Vagina thick, cylindrical, longer than free oviduct. Basal part of diverticulum thick, thinning to apical part and attached to spermoviduct. Spermoviduct thick and long. Pedunculus of bursa copulatrix slender and long. Bursa copulatrix large, oblong.

#### Diagnosis

Shell fusiform, thick, solid, opaque, reddish-brown, height 24.7–27.5 mm, with 10.5–12.25 whorls. Peristome strongly doubled, thick, expanded and reflected, with or without indistinct palatal serration. Inferior and subcolumellar lamella close and weak, the former descending to near the peristome margin and the latter descending to the peristome margin. Palatal plicae 4–5, short, lateral, nearly straight or slightly curved. The new species can be easily distinguished from *M. takagii* and *M. pretiosa* by the fusiform shell (vs. slender). It is similar to *M. gregoi* by the similar shell shape, but differs by the larger shell (shell height 24.7–27.5 mm vs. 15.6–18.9 mm), the stronger first peristome, the stronger subcolumellar lamella and the closer position of the two peristome margins (Fig. 1).

#### Etymology

The specific name is made from the Pīnyīn form for China West Normal University, the alma mater of the first author. Next year will be the 80th anniversary of the university's founding and the first author wishes to commemorate this with the species name. The vernacular name is 西华师大奇管螺 (Pīnyīn: xī huá shī dà qí guǎn luó).

#### Distribution

**Figure 3.**
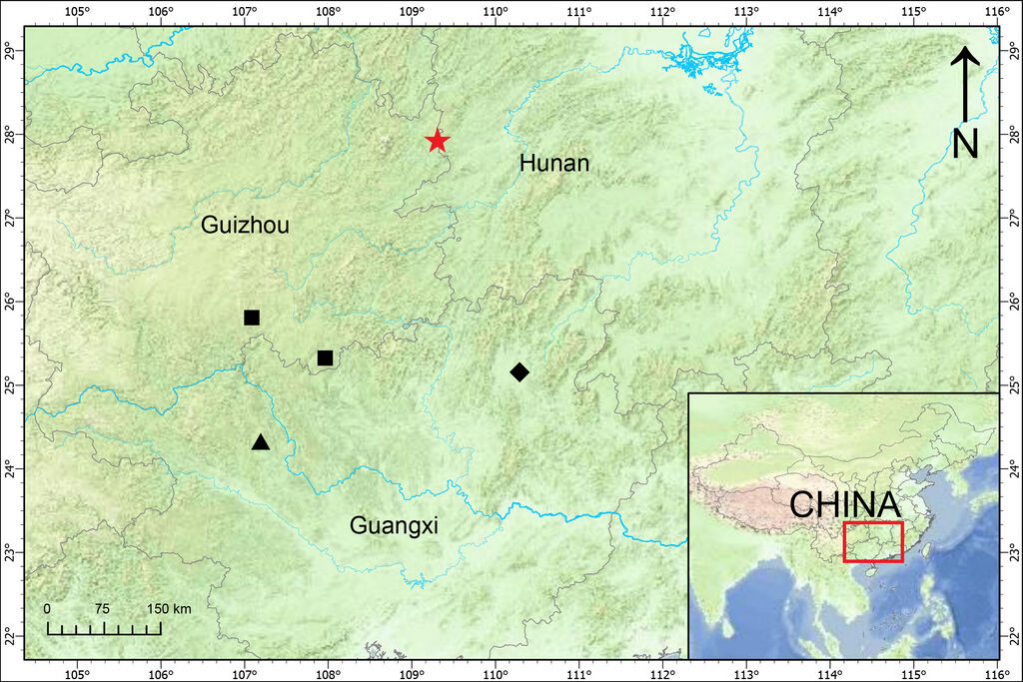
Distribution of *Miraphaedusa* species. Star: *Miraphaedusa xihuashida* Chen, sp. nov., diamond: *M. takagii*, triangle: *M. pretiosa*, squares: *M. gregoi*.

Known from the type locality only (Fig. 3).

#### Ecology

Living animals were found under fallen leaves in dense shrubs together with *Cyclophorus* sp. and *Bradybaena* sp.

#### Taxon discussion

Nordsieck (2012a) treated the monotypic genus Falsiluna Grego & Szekeres, 2011 as a subgenus of Miraphaedusa. Subsequently, Hunyadi and Szekeres (2016) restored the independence of *Falsiluna*. *Falsiluna harryleei* Grego & Szekeres, 2011, the only species of of its genus, has dextral shell with lunella-like palatal plicae, which strongly differ from those of *Miraphaedusa*. Therefore, we treat *Falsiluna* as an independent genus, while *Miraphaedusa* contains only three species and *M. xihuashida* Chen sp. nov. The new species occurs in north-eastern Guizhou, far away from the localities of its congeners (Fig. 3).

## Supplementary Material

XML Treatment for Miraphaedusa xihuashida

## Figures and Tables

**Figure 1a.**
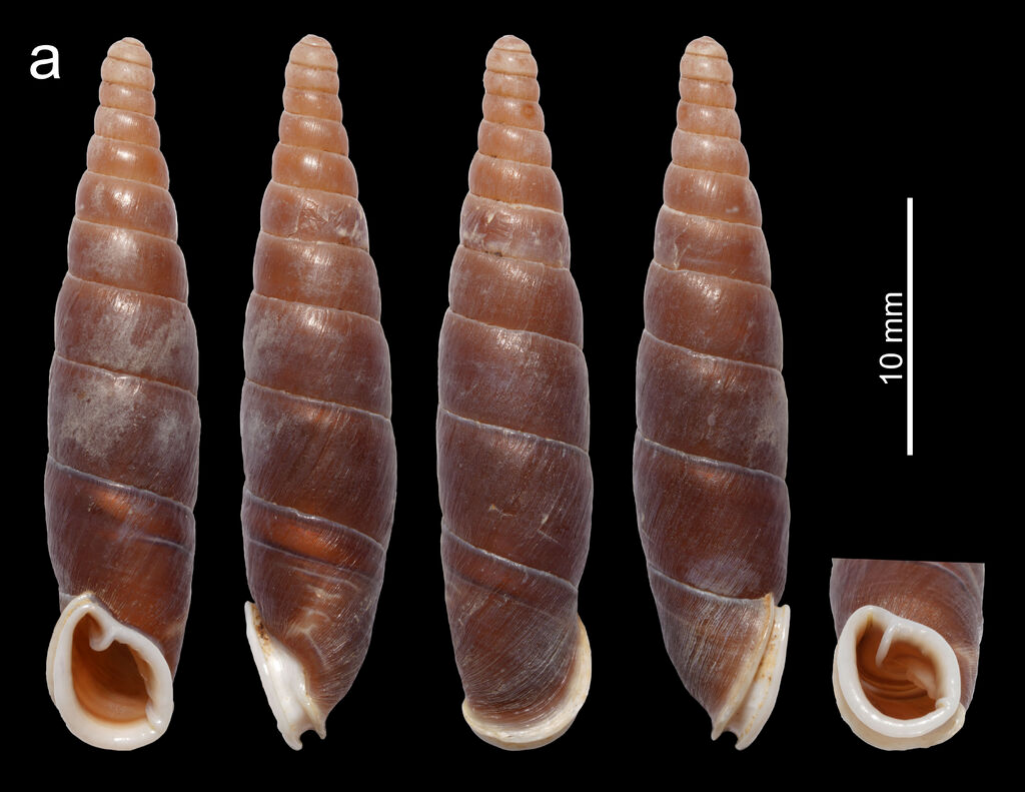
*Miraphaedusa xihuashida* Chen, sp. nov., holotype;

**Figure 1b.**
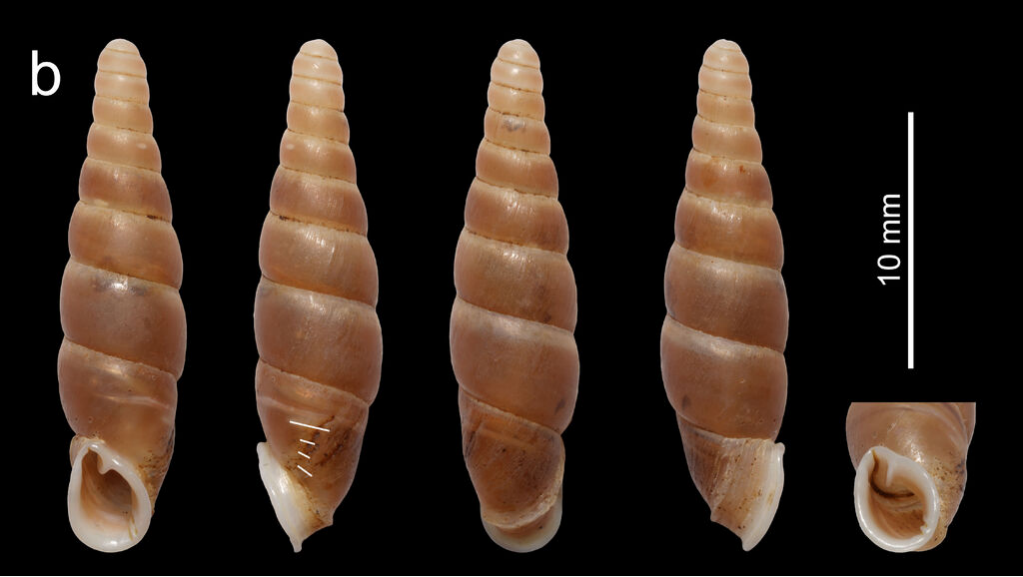
*M. gregoi*, Yanzidong, Huishui, Guizhou, China.
